# Business as usual? Assessing the impact of the COVID-19 pandemic to research, development and innovation (RDI) activities of universities of applied sciences

**DOI:** 10.1007/s11233-021-09079-z

**Published:** 2021-11-03

**Authors:** Maria Salomaa, Andrea Caputo

**Affiliations:** 1 External Funding, Tampere University of Applied Sciences, Kuntokatu 3, 33520 Tampere, Finland; 2 Lincoln International Business School, University of Lincoln, Lincoln, UK; 3 Department of Economics and Management, University of Trento, Trento, Italy; 4 Department of Management, University of Lincoln, Lincoln, UK

**Keywords:** Innovation, RDI services, Case studies, Remote working, Knowledge transfer, COVID-19

## Abstract

Universities of applied sciences (UAS) have a strong mandate to carry out research, development and innovation (RDI) activities in collaboration with local stakeholders. Geographical proximity is one of the key factors for the creation and success of RDI activities because of the positive balance between costs and benefits of local knowledge transfer, but they also depend on the networks of individual staff members. This paper aims to explore how maintaining and developing purpose-built and individual RDI partnerships during the COVID-19 pandemic has been managed. An enhanced conceptual framework for assessing contextual dimensions of the RDI activities beyond academic entrepreneurship as business ventures has been developed. The paper focuses on a single case study drawing on semi-structured research interviews investigating how the swap to remote working have affected RDI activities at Tampere University of Applied Sciences, one of the biggest UAS in Finland with intense regional linkages. The contribution of the study is twofold; firstly, the paper introduces an expanded theoretical approach for assessing the external and internal factors having an impact on the RDI activities beyond academic entrepreneurship. Secondly, by testing the proposed framework, it shares insights and good practices derived from empirical evidence, namely binary data and semi-structured interviews revealing experiences of RDI personnel and project managers involved with different phases of RDI process, for optimising high-quality innovation support, knowledge transfer activities and co-creation of new knowledge in exceptional circumstances.

## Introduction

Started in late 2019 in China, and spread globally during the early months of 2020, the COVID-19 pandemic is disrupting life due to the high infection rate, which is forcing institutions and societies to rethink how to coexist with an unknown virus until a vaccine or effective treatment is found. Among many other actors, research institutions and universities are severely affected by a pandemic that is radically changing the three main missions of such entities, namely education, research and engagement. Campus closures, travel restrictions, social distancing and lockdown measures are causing a quick shift towards online teaching, learning and adapting to remote working practices. The impact is perhaps more severe on the research and engagement activities, in which the social and institutional network of relationships and knowledge exchanges must happen virtually. The combination of such situations asks for new knowledge on how research institutions are coping with the pandemic, and what kind of impact it has on the research, development and innovation (RDI) activities. In this study, RDI activities are understood mainly through university-business collaborations, which entails different kinds of formal and informal ‘cooperative interactions’ seeking to generate mutual benefit (Davey et al., 2011) often linked with h the traditional university core missions of teaching and research (Galán-Muros & Plawa, 2016).

Policy-makers have come to regard local higher education institutions as one of the key actors of regional development (e.g. Goddard & Vallance, 2013; Breznitz & Feldman, 2012). Thus the higher education institutions are currently expected to respond to various needs of external stakeholders (Uyarra, 2010). For the past decade, their regional role has been emphasised also in the policy design processes, in which the smart specialisation approach dominating the implementation of the EU Cohesion Policy (e.g., Begg, 2016) have tied universities closer to the Research and Innovation Strategy for Smart Specialisation (RIS3) formulation and implementation (e.g. Charles et al., 2014). Despite the emerging policy pressures and other external factors urging universities to adapt a so-called ‘one-size-fits-all’ model towards the engagement activities, in practice universities “create their own approaches and models of the third mission by targeting different areas of activities, partners and geographical areas, and by combining different set of missions, capabilities and resources.”(Kitagawa et al., 2016, p. 744). The heterogenous institutional responses towards the university engagement call for further research on the interactions that take place between specific regions and characteristics of universities (e.g. Wright, 2014).

In practice, many businesses have a tendency to prioritise cooperation with local universities for various reasons, e.g. easy access to university knowledge, short-term benefits, lower knowledge transfer costs and contributions to local community (e.g. Fitjar & Gjesvik, 2018). In some way this implies that proximity is one of the factors increasing collaboration between universities and local firms; yet, it has been argued that the mere presence of higher education institutions does not ensure that knowledge transfer will happen (e.g. Arnkil et al., 2010). There is also some evidence that the geographical proximity alone does not explain what kind of partnerships generate innovation; instead of serendipitous, casual encounters, innovation is driven by purpose-built relationships (Fitjar & Rodríguez-Pose, 2017). Thus, one of the questions that remains open for further investigation is related to how a particular location can be related to the university’s innovation activities and its role in the business ecosystem (e.g. Wright, 2014) as the theoretical knowledge base on the development of successful university-industry linkages and their management remains rather limited (Plewa et al., 2013). This calls for both conceptual and empirical studies revealing the success factors of the university engagement and the changing modes of stakeholder interactions and business models (Miller et al., 2014).

In the context of Finnish Higher Education, research, development and innovation activities are at the core of ‘universities of applied sciences’ (UAS) activities, a characteristic which places such institutions in a particularly favourable position for offering a relevant context for empirical studies. They have a strong base to carry out RDI activities with local stakeholders, and compared to traditional research universities, the UASs are more likely to adapt managerialist practices (Aarrevaara et al., 2011) to drive business collaboration. However, the UAS’s working life linkages have been described rather as bottom-up initiatives instead of results of systematic development of institutional bridging mechanisms (e.g. Maassen et al.*,* 2011). This means that the RDI activities build upon staff members’ individual networks and active student participation to collaborative projects and initiatives, rather than stemming from an active strategic direction. Thus, the UASs are important promoters of innovation in group-based and networked learning environments in Finland, and key actors in regional development (Kettunen, 2011). This means they are particularly at risk of the disruption brought by the COVID-19 pandemic that may alter the regional development landscape of such institutions, resulting in a long-term damage to knowledge transfer activities and economic development of their local communities.

This paper aims to investigate, in the context of the Finnish universities of applied sciences, how relationships with external partners can be managed, maintained and developed in the time of COVID-19 crises. When proximity is replaced by collective social distancing, what are the key mechanisms to support and drive local innovation? What kind of tools and managerial practices can facilitate the success of these collaborative activities when working remotely? To contribute to finding answers to such questions, a single case study of Tampere University of Applied Sciences (TAMK), one of the biggest UAS in Finland with intense working life connections within the Tampere region and beyond, was chosen to investigate how remote working have affected to their RDI activities. This was done by analysing the impact of the COVID-19 pandemic to the four contextual dimensions of academic entrepreneurship originally conceptualised by Wright (2014). Although further studies are needed, the findings indicate that higher education institutions with a strong RDI capacity may be less affected by major changes, such as pandemics, having an impact to the whole operational environment. In the case of TAMK, strong personal linkages and tradition of business collaboration have even led to new academic ventures demanded by the changes in the market. The contribution of the study is twofold; firstly, the paper introduces an expanded theoretical approach for assessing the external and internal factors having an impact on the RDI activities beyond academic entrepreneurship. Secondly, by testing the proposed framework, it shares insights and good practices derived from empirical evidence, namely binary data and semi-structured interviews revealing experiences of RDI personnel and project managers involved with different phases of RDI process, for optimising high-quality innovation support, knowledge transfer activities and co-creation of new knowledge in exceptional circumstances.

## RDI activities within the UAS – driving local innovation remotely?

### RDI activities within the Finnish UAS

Finnish Universities of Applied Sciences are public organisations steered by national higher education policies. They have an explicit task to engage and support their regional stakeholders (Act 932/2014) through applied research and other research, development and innovation activities aiming towards ‘regional cluster development’ (Melin et al., 2015). It is a shared strategic aim of the Finnish UASs’ RDI activities to serve their communities and “to produce information and develop services based on applied research that serve to strengthen the competences and competitiveness of the public agencies and private business community in its region.”(Maassen et al., 2011, p.20). This is done whilst linking education to RDI activities to ensure that the future graduates are equipped with skills and competencies responding to the actual needs of the employers (Heino, 2017). Previous studies have argued the UASs respond better to local employment demands than research universities, increasing their ability to implement the university third mission successfully (Jaeger & Koppler, 2014). These external linkages have become even more important during the COVID-19 pandemic as HEIs are one of the key actors taking part in public debate on the pandemic, sharing multidisciplinary knowledge on various aspects of the crises for policymakers, generating digital solutions and financial forecasts. This further increases the regional role of universities, in particular in ‘crisis management’ and knowledge transfer (Fitjar, 2020).

Despite many reforms in the Finnish higher education system, based on dual model since the early 1990s, the UASs’ task to act as a link between the university research and its application to the public and private sectors has remained somewhat vague (Melin et al., 2015). However, as Galán-Muros and Plawa (2016) argue “if activities in the research and valorisation domains become more defined, objective and transparent, the current barriers [of university-business collaboration] might reduce or disappear.”(p. 378). Indeed, there is an ongoing discussion on the research mandate of these ‘non-university institutions’ (Teichler, 2008) including polytechnics, university colleges as well as the UASs (Elmgren et al., 2016). Overall, the UASs’ traditional task in the higher education binary system to offer high-quality professional and vocational education on the BA level without an explicit research mandate have become blurred (Taylor, 2008). In Finland, this has been explained by a lack of a political steering framework, state funding, suitable funding instruments, legal support, and strong orientation towards education (Maassen et al., 2011). This aligns with the well-known limitations of top-down policy implementation, particularly in regard to science and technology transfer, as they depend on organisational routines of the implementing institutions (Hellström et al., 2017). Also the UASs have traditionally nurtured bottom-up approaches towards developing institutional RDI profiles, which range from well-connected regional actors to UASs with weaker linkages between research and RDI (Kajaste, 2018).

### The contextual dimensions of the university’s RDI activities

Academic entrepreneurship and technology transfer are typical approaches for investigating (economic) engagement activities conducted by higher education institutions (e.g. Grimaldi et al., 2011). Along with the increased focus on the impact of changing and complex environments highlighted in both institutional and entrepreneurship studies (e.g. Welter & Smallbone, 2011; Welter et al., 2019), the innovation policy and other external factors can also shape the universities’ research, development and innovation activities. Thus, instead of a narrow conceptualisation of ‘academic entrepreneurship’ as spinoffs and other business ventures, a wider perception on the university’s institutional resources and capabilities as well as the impact of the innovation ecosystem in generating ‘sustainable returns’ have been called for (e.g. Wright, 2014). Therefore, in this study, the university RDI activities are understood rather broadly as different kinds of university-business collaborations. This can entail many kinds of formal and informal ‘cooperative interactions’ between universities and businesses seeking to generate mutual benefit (Davey et al., 2011), which are strongly associated with the core missions of teaching and research (Galán-Muros & Plawa, 2016).

Wright’s conceptualisation of academic entrepreneurship (2014) – based on Zahra’s and Wright’s (2011) previous work – is one among the more recent attempts to capture the heterogeneity of university-business collaborations and its implications on both micro and macro level. Wright identified four contextual dimensions having on impact on the resource orchestration of academic entrepreneurship, namely temporal, institutional, social and spatial dimensions, which are next further discussed reflecting their relevance to the RDI activities of higher education institutions.

In Wright’s conceptualisation of academic entrepreneurship, *the temporal context* refers to the emergence and life-cycle of academic spin-offs (Wright, 2014), that can be defined as “a start-up created when the licensee of a university-assigned invention creates a new company to exploit it” (Di Gregorio & Shane, 2003, p. 2010). The academic spin-offs are considered as one of the manifestations of the ‘third mission’ through which the university contributes to local economic development (Philpott et al.*,* 2007). However, the discussion on the academic spinoffs also entails different forms of knowledge transfer to introduce innovation ultimately leading to job-creation as well as more unquantifiable methods of knowledge exchange and co-creation involving both public and private actors (e.g. Göransson et al., 2009) and mechanisms to support academic ventures from TTO’s to entrepreneurship education (Sansone et al., 2019). The temporal context to ‘academic spinoffs’ thus requires sufficient human, financial, technological and networking resources to pursue ventures from academic spill overs (Wright, 2014), but also a range of targeted support mechanisms to foster business-collaboration as well as entrepreneurial capacities of the staff and students (Urbano et al., 2017).

*The institutional context* refers to government policies steering universities’ innovative activities (Wright, 2014). In practice, this entails both higher education and innovation policies, which can, to a certain extent, have opposing goals: whilst higher education policies drive research excellence (e.g. Goddard & Vallance, 2013), the regional policy-makers regard higher education as an important engine of economic growth (Breznitz & Feldman, 2012). The policy push towards third mission has broadened the scope of universities and made them ‘organizational umbrellas’ for different tasks from scholarship to entrepreneurial activities (Wildavsky, 2010), and universities are expected embed economic and social development to the core functions, combining research, teaching and technology transfer (Etzkowitz et al., 2008). In addition, (local) RDI funding instruments and regional engagement are among the key factors in changing the orientation of higher education (Gibb & Hannon, 2006) towards academic entrepreneurship. As Zahra and Wright (2011) state, the policymakers should thus reflect what kind of entrepreneurship they want to foster, especially the notion of innovation have shifted towards more broader conceptualisations (Wright, 2014).

*The social context refers* to the overall business ecosystem in which the university and academic entrepreneurship operates. The social context can vary in sectors and disciplines having an impact on the development of innovations, types of knowledge transfer and opportunities for academic ventures (Wright, 2014). It has indeed been suggested that overall (economic) operational environment shapes the way in which the university carries out engagement activities (Foss & Gibson, 2015), including business collaboration, knowledge transfer mechanisms, supporting entrepreneurship and exploitation of academic knowledge spill overs (e.g. Salomaa, 2019). In practise, this could entail providing tailored innovation support or purpose-built study programmes targeted to respond to the needs of local businesses or regional priority sectors. These kinds of customised activities are required especially when the social context is disrupted by global challenges having an impact on the market, business landscape and demand.

*The spatial context* relate to the specific location in which the academic entrepreneurship takes place as well as mobility issues; whilst the university is spatially fixed, the academics tend be highly mobile (Wright, 2014). Previous studies have explained the differences in universities adaptations of the engagement activities partly by geographic factors (e.g. Kitagawa et al., 2016). Although firms may have a tendency to prioritise cooperation with local universities for easy access to university knowledge, short-term benefits, lower knowledge transfer costs and contributions to local community (e.g. Fitjar & Gjesvik, 2018), it is not given that a mere presence of a higher education institution initiate knowledge transfer or other business collaboration (e.g. Arnkil et al., 2010). On the contrary: it has been claimed that instead of geographic proximity the most successful partnerships driving innovation are based on purpose-built relationships (Fitjar & Rodríguez-Pose, 2017), which emphasises the temporal context facilitating (academic) business ventures by incorporating entrepreneurship to research and teaching. As Wright (2014) states, the identification of different roles played by universities in different spatial contexts is also a question of institutional context in the sense that suitable policies for driving innovation in different regional contexts are needed.

### The impact of COVI-19 pandemic to the university RDI activities - driving innovation local innovation remotely?

Maassen et al. (2011) detected, that there is a lack of systematic bridging mechanisms between the Finnish UASs and external partners, including both public and private organisations, partly explained by the absence of structured evidence of RDI outputs and insufficient communication: “--it is often unclear for companies what exactly the UAS expertise is and how it can benefit their company strategy.”(p.22), which can be related to temporal, institutional and social contexts of academic entrepreneurship. Also Heino (2017) identified, that it is indeed challenging “to make the expertise and facilities easily available to companies and public organizations of the region to improve their RDI capability.” (p.105), which refers mostly to internal communication issues within universities. Furthermore, it can be complicated to conduct RDI activities ‘in a professional manner’, which could be facilitated by having a small group of RDI specialists with a cumulative expertise to deal with administration and financial aspects of different public funding instruments (Heino, 2017). Whilst these RDI support units mainly concentrate on the bidding processes and administration, the majority of the research and development activities actually take place in more educational units (Kajaste, 2018). The role of research support services can be crucial in establishment of the contacts and bidding processes, but the rapid leap to remote working has made communication more challenging. This urges HEIs to become innovative in developing new ways to contribute to their region, especially in the exceptional circumstances derived by the COVID-19 pandemic (Fitjar, 2020), which has not only urged universities to quickly adapt remote teaching methods, but also to reinvent networking and communication tools for both maintaining and initiating collaborative actions with external partners.

As previous studies indicate, the organisation’s capacity to drive innovation depends on internal capacity to manage different task for promoting collaboration and creativity, also among remote workers (e.g. Silva & Merino, 2017), emphasising proactive capacity to initiate RDI activities (temporal context). Thus the quick adaptation to remote work because of the COVID-19 pandemic may affect to employees in many ways. Whilst remote working allows greater autonomy and decreases micromanagement, creates a sense of trust and increase individual’s commitment to the organisation, there can also be increased feeling of isolation, which requires more attention to maintaining interpersonal relationships, creation of social support networks and good coordination of online work activities (e.g., Charalampous et al., 2019). We therefore seek to study the impact of the pandemic to the university RDI activities through different contextual dimensions of academic entrepreneurship presented in previous section. A case of Tampere University of Applied Sciences for its strong regional focus and success in implementing an efficient entrepreneurship strategy (Seikkula-Leino & Salomaa, 2020). The pandemic have undoubtedly caused changes in all four dimensions related to academic entrepreneurship, but especially the social context has been disrupted. This particularly relevant for HEIs, being that their business engagement largely rely on personal relationships (e.g. Davey et al., 2011; Plewa et al., 2013). Have the HEIs been able to assist regional partners in responding to the challenges emerged from the current COVID-19 crisis? How the connections between external partners have been maintained and developed through the crises and how this have affected current and future RDI activities?

## Methods and materials

A case study approach was employed for assessing the impact of the COVID-19 crises to the RDI activities of the Finnish UASs as it is particularly suitable method for creating deeper understanding on the phenomenon (Saldaña, 2011; Flyvbjerg, 2006). It is also a well-suited approach for understanding the uniqueness of a particular context (Patton, 2015; Saunders et al., 2016), but the findings can be valuable to the wider higher education community also beyond Finland, in particular for regionally-oriented universities and countries with a dual HE system, such as Denmark, Ireland, the Netherlands and Switzerland.

Following the orders of the Prime minister, the Finnish HEIs closed their campuses on the 18th of March 2020. Since then, the majority of their personnel have worked remotely until further notice. The case HEI, Tampere University of Applied Sciences (TAMK), one of the Finland’s 22 UASs, was chosen because of its dense working life connections and strategic aim to increase the volume of RDI activities. TAMK has already reinforced its organisational capacity for the latter e.g. through four newly established research groups.^1^ Considering the recent merger of the former Tampere University of Technology and the University of Tampere, and especially TAMK’s role in the new Tampere University Community, TAMK represents a unique case in the Finnish UAS scene; such ‘atypical cases’ are ideal for obtaining richer data sets and they enable in-depth investigation of the phenomenon in question (Flyvbjerg, 2006).

TAMK is a multidisciplinary UAS consisting 13.000 students and it offers a wide range BA and MA degree programmes focusing on health and wellbeing, business and technology. Its annual budget is over 65 ME. TAMK’s current mission statement brings the working life connections and the RDI activities to the centrefold: “Our strong orientation towards working life ensures the best learning possibilities for our students. Furthermore, we are involved in research, development and innovation which specifically target the development needs of working life.”^2^ TAMK has also set three impact areas, in which the business and industry collaboration are further reinforced.

This study draws on semi-structured interviews conducted with TAMK’s personnel involved with different phases of the RDI processes – management, planning and implementation – from both education units and RDI service unit. Altogether, ten phone and one email interview were carried out in May 2020. The total length of all conducted interviews was 5 h and 13 min. At the time of the interviews, the participants had worked remotely between six to nine weeks. The interview recordings together with lead author’s notes from the interviews form the base for the thematic analysis following the logic of the conceptual framework proposed in previous section (Table 1). In order to disseminate accurate findings in a timely manner in the midst of the COVID-19 crises, the member checking approach (Geertz, 1973) was employed and all interviewees were given the opportunity to comment this paper to increase validity and reliability of the study before the submission of the draft.

**Table 1:** Conceptual framework for assessing four dimensions of academic entrepreneurship related to RDI actvities

Context	Dimension	Estimated relevance for the university RDI activities
Temporal Context	The emergence of (academic) spin-offs; Life-cycle from opportunity to sustainability	Proactive RDI capacity; (Organisational and individual) human capital, financial and technological capacity as well as networking resources for supporting academic ventures from spinoffs to other university contributions towards sustainable economic development.
Institutional (policy) context	Government policy towards universities; innovation system	RDI goals: Higher education and innovation policies steering towards entrepreneurship and defining conditions for (business) engagement activities (e.g. funding instruments).
Social context	Business ecosystem	Market demands for RDI: The overall business ecosystem (sectors, business landscape, networks) in which the university drives academic entrepreneurship steers towards disciplinary choices and has an impact on the type and volume of university-business collaboration.
Spatial context	Geographical location	Spatial demands for RDI: The specific location in which the academic entrepreneurship takes place has an impact on opportunities and demand for university-business collaboration.

Source: Authors’ elaboration based on Wright (2014)

## Findings and discussion

### Key findings

According to all interviewees, TAMK had a good organisational capacity to adapt quickly to remote working in all its operations. The RDI support staff members, involved with both pre and post-award phases, agreed that they have been able to carry on their daily tasks remotely with online tools (e.g. Microsoft Teams, Zoom). Overall, the implementation of the ongoing RDI projects runs smoothly, although online meetings tend to be very structured, leaving less room for discussion and exchanging of innovative ideas for future RDI activities. Also, the funding authorities have been flexible and quick to react to questions related to implementation of projects in exceptional circumstances.

The interviewees agreed that the impact of the remote working followed by the global COVID-19 pandemic has been marginal, but some challenges have emerged. These challenges very related to all four contextual dimensions of the academic entrepreneurship having an impact to the RDI activities. In the following sections, these key findings, varying from minor IT problems to more extensive challenges related to external partners’ limited organisational resources to work remotely (including international partners involved with of Erasmus+ Capacity building projects) as well as substantial organisational issues related to communication, networking with business partners and overall working culture towards RDI activities, as discussed in detail. Finally, the observed impact of the COVID-19 pandemic to the case university’s RDI activities is summarised in Table 2.

**Table 2:** Assessment of the observed impact of the COVID-19 pandemic to university RDI in the case of TAMK

Context	Dimension	Estimated relevance for the university RDI activities	Observed impact of the COVID-19 pandemic in the case of TAMK
Temporal Context	The emergence of (academic) spin-offs; Life-cycle from opportunity to sustainability	Proactive RDI capacity; (Organisational and individual) human capital, financial and technological capacity as well as networking resources for supporting academic ventures from spinoffs to other university contributions towards sustainable economic development.	Strong organisational capacity to swift to remote working; Selected RDI project activities delayed until after the crises (e.g. seminars and workshops demanding physical presence); Teachers burdened with remote teaching, whilst management concerned about increased volume of new project ideas from the educational staff; Internal communication less effective; No specific managerial procedures for ensuring volume and quality of RDI during the crises; Decreased opportunities for serendipitous encounters leading to innovative project ideas.
Institutional (policy) context	Government policy towards universities; innovation system	RDI goals: Higher education and innovation policies steering towards entrepreneurship and defining conditions for (business) engagement activities (e.g. funding instruments).	Strong encouragement towards COVID-19 specific calls; Participation in policy design processes related to targeted funding schemes to tackle the crises and mitigate its economic and societal impact (e.g. Structural Funds schemes)
Social context	Business ecosystem	Market demands for RDI: The overall business ecosystem (sectors, business landscape, networks) in which the university drives academic entrepreneurship steers towards disciplinary choices and has an impact on the type and volume of university-business collaboration.	Collaboration with business partners slower (e.g. furloughs of the key staff members; teachers burdened with remote teaching); Regional business contacts established and maintained through dedicated staff members; New multidisciplinary RDI activities emerging from the collaborative initiatives to mitigate the pandemic (e.g. Licence to Breathe).
Spatial context	Geographical location	Spatial demands for RDI: The specific location in which the academic entrepreneurship takes place has an impact on opportunities and demand for university-business collaboration.	Increased collaboration within the Tampere University Community (e.g. collaborative actions towards Business Finland); Virtual meetings as the ‘new normal’ enable including new partners more easily to planning processes; Personal linkages have become more emphasized.

The temporal context reflecting the proactive RDI capacity of the university was described to be slightly disrupted by the remote working. The interviewees observed that the communication within the RDI service unit and between TAMK’s educational units have decreased after shifting to remote working, slowing the flow of knowledge within the organisation. This might create significant knowledge gaps related to new RDI projects and calls, which are both important information for staff members dealing with RDI issues. More systematic internal communication would increase the sense of belonging and facilitate team building. As an example, the RDI unit’s daily ‘virtual café’ meetings were considered to be important, but unsuitable channel for circulating information on the current RDI issues; as participation is not mandatory, team members might miss relevant information. This could be decreased by systematically developing tools for effective communication to ensure correct flow of information. In addition, supportive messages from the top management – instead of just sharing pragmatic guidelines referring to efficient use of granted RDI funding to ensure timely completion of the ongoing projects – would help personnel to adjust to the new mode of working. Although the staff members are able to work very independently, especially the personnel involved with the implementation of the RDI projects hoped for a clearer communication and that the management would explicitly express how they are ready to support the staff during the pandemic.

Considering the future RDI activities, the interviewees had mixed opinions on the impact of the COVID-19 to TAMK’s business collaborations, which represents the social context steering the type and volume of cooperation. Despite the urgent need to develop new operational models, according to the interviewees, the businesses working in the affected sectors are overloaded with running their day-to-day operations, either because of the increased/decreased market demand and / or lack of human resources following furloughs. Some staff members estimated, that TAMK has insufficient organisational capacity to maintain and develop purpose-built business collaboration and networks, which is even more evident in the time of crises: the staff members needed to adapt to remote teaching in a very short notice, and these re-arrangements were prioritised over RDI activities to deliver high-quality education despite the exceptional circumstances. Nonetheless, the majority of the business contacts depend on personal linkages between the teaching staff members and companies, whilst the RDI support services have a somewhat smaller role in building and maintaining these connections. The latter, however, has established a dedicated team focused on networks. In both cases, the contacts are traditionally established in face-to-face meetings. During the pandemic, the existing personal contacts with business partners have become crucial, especially in designing new projects. Overall, the remote working was considered to be easier with people that already had at least some sort of history of collaboration, whilst involving ‘newcomers’ to the planning processes could create tensions and even hinder designing of the projects. This can be facilitated by employing virtual platforms (e.g. Miro) to large-scale planning processes. However, planning and networking – especially with international partners – will become more problematic in the long-term, as virtual platforms and webinars are more focused on knowledge transfer than creating serendipitous encounters.

The interviewees estimated, that the actual impact of the COVID-19 pandemic to different sectors will develop on the long-term, and many businesses are still working ‘as usual’ (e.g. construction and built environment). However, some changes were already observes in the institutional (policy) context, such the emergence of targeted funding schemes to increase university-business collaboration to mitigate the economic and societal impact of the crises. In practice, the pandemic had created new opportunities within the Tampere Higher Education community and created new interaction also beyond the higher education sector, in particular in disciplines put in the centrefold in tackling of the crises (e.g. indoor hygiene, digitalized health services), which suggests that the urgency to find new solutions increases collaboration in the spatial context. On a regional level, one of the key actors to ensure interaction with local companies within the Tampere region are TAMK’s dedicated regional representatives, who have been directly in contact with businesses to promote TAMK’s expertise to the companies during the pandemic.

On the management level, the Finnish UASs have been actively feeding themes to future Structural Funds (SF) calls to tackle the COVID-19 crisis, demonstrating proactive commitment towards shaping the institutional (policy) context steering the RDI goals for the HEIs. Indeed, many of the final national and regional level SF calls of the programme period 2014–2020 have a specific focus on the mitigation of the economic and societal impact of the COVID-19 pandemic (e.g. increased unemployment, social exclusion). In addition, TAMK has reacted to the COVID-19 specific calls (e.g. Business Finland) with business and university partners, which has led to multidisciplinary collaborative projects aiming to tackle the challenges posed by the coronavirus. One of them is called ‘License to Breath’, which is a project for examining the indoor transmission of the coronavirus through surfaces and ventilation systems in collaboration with Faculty of Built Environment and the Faculty of Medicine and Health Technology at Tampere University and from Tampere University of Applied Sciences. The team includes experts in medicine, biosciences, fluid mechanics, aerosol physics, building services engineering as well as the property sector.

### Discussion: the impact of the COVID-19 pandemic to RDI activities in the framework of academic entrepreneurship

The findings show how a regionally-focused higher education institution, such as the Finnish Universities of Applied Sciences, has a good strong expertise to carry out RDI activities (temporal context) and knowledge on the central funding instruments, which can facilitate businesses without previous experience to engage with publicly funded RDI projects. This demonstrates a proactive approach towards the policy context and demonstrates how the (local) RDI instruments can change the (regional) orientation of higher education (Gibb & Hannon, 2006). Furthermore, an active role in the implementation of the projects can shape the social context by responding to the special needs of the local companies (Foss & Gibson, 2015) – in the case of TAMK, this was more evident through the university’s role in facilitating access to public funding. The management indeed regarded the targeted COVID-19 calls as central instruments for HEIs to support local businesses, but otherwise there were no specific top-down guidelines or recommendations how to carry on RDI work remotely. Also the personnel working on the RDI project design agreed, that although business collaboration is slower in the time of the crises, the ‘COVID-19 calls’ provide opportunities to conduct R&D with a strong, evidence-based demand from the society. These initiatives could lead to long-term collaboration, even when the project partners are contacted ‘only’ remotely; the digitalisation of the meetings as a ‘new normal’ enable including new partners to the virtual planning processes more easily. These new ways of online working can thus reduce the importance of the spatial context, as some of the typical benefits of cooperating with local universities, such as easy access to university knowledge and lower knowledge transfer costs (Fitjar & Gjesvik, 2018) may become irrelevant.

Whilst the Finnish UASs have become more focused on RDI activities in the past decade, their research mandate remains vague (Taylor, 2008) and the overall working culture is strongly oriented towards teaching, which can affect all dimensions of the academic entrepreneurship. In the case of TAMK, this was described to be a bigger barrier for RDI activities than the swap to remote working; education is the central line of activity, and there are partly insufficient organisational mechanisms to support RDI activities of the teaching staff (e.g. resource allocation, delayed target setting for RDI, inexperienced project managers). This is aligned with Urbano et al.’s (2017) notion, that both support mechanisms of business-collaboration and fostering entrepreneurial competencies of the staff and students are needed to reinforce the university’s proactive RDI capacity.

To conclude, the role of internal communication cannot be exaggerated in making the temporal context more effective. In the case of TAMK, the management was concerned about the decreased amount of RDI initiatives emerging from the educational units during the crises, but the statistics obtained from TAMK’s internal project management tool revels that the cumulative amount of new RDI initiatives going through the internal evaluation process between January and September 2020 (118 submitted proposals) is actually equivalent to the situation from January to September 2019 (120 submitted proposals).

## Conclusion

This study sought to examine how the swift to remote working following the COVID-19 pandemic have changed the research, development and innovation activities within a regionally-focused university, belonging to the group of the Finnish Universities of Applied Sciences. In this paper, we proposed and tested an enriched framework based on Wrigth’s (2014) conceptualisation of different dimensions of academic entrepreneurship to assess the impact of the pandemic to the university RDI activities. Yet, the study has several limitations. The first and main research limitation consists in the adoption of a single case study as a methodological approach, which allowed for in-depth analysis but may raise concerns of generalisation of findings. However, we mitigated such limitations by anchoring our investigation to a rich and solid theoretical framework based on previous studies on the university engagement. Another limitation may lie in the regional element of the case study as findings may be influenced by cultural, contextual and technical determinants pertinent to Finland and the Tampere region, and as such, should be read and interpreted within similar contexts. Future research efforts could fill such gaps either through the adoption of alternative methods, e.g. quantitative methods or by replicating single or multiple case studies to develop additional fine-grained insights, from Finland and beyond. This would enable producing for a growing pool of data for eventually achieving a wider generalisability (Cohen et al., 2017).

Nevertheless, albeit from a single case study, our findings from Tampere University of Applied Sciences suggest that the UASs have a good organisational capacity to carry on RDI activities remotely. In the time of the crises, tacit knowledge has become highly valuable in the preparation of new RDI initiatives. Although the virtual meetings as a ‘new normal’ enable including new, often distant, partners to the design processes, they can create internal knowledge gaps and increase the sense of exclusion among the staff members (e.g., Charalampous et al., 2019). Therefore, more effective internal communication and clearer tasks within the organisation, as well concerning the overall role of the university-business-collaboration within the higher education institutions (Galán-Muros & Plawa, 2016), would facilitate internal knowledge flow and more smooth designing of new projects in order to reach the organisational vision to provide high-quality RDI services (Heino, 2017). Also, a more systematic development of purpose-built engagement mechanisms are needed to enable maintaining long-term collaboration with external partners beyond the crises, as currently the networks often rely on personal linkages and personal relationships (‘weak ties’) (Davey et al., 2011; Plewa et al., 2013) and decentralised business-connections. In the case of TAMK, the UASs explicit task to support their regions was carried out by the dedicated regional representatives, but the overall organisational capacity to react quickly to the needs of the companies relies on largely the targeted funding schemes to tackle the crises and mitigate its economic and societal impact (e.g. Business Finland, Structural Funds schemes).

These findings suggest that, in the time of the COVID-19 crises, everything that has worked well before in regard to higher education institutions RDI activities, still works. The results from the case study did not reveal any major interruptions caused by the pandemic followed by social distancing and remote working. Thus the proactive RDI capacity (temporal context) of the organisation seems to be a defining factor in how they cope in the midst of the crises: the readier it is to carry out RDI tasks, the less its RDI activities are affected by changes in the institutional, social and spatial contexts. Therefore, further promotion of RDI activities among the key operations of the UASs would help to develop the current, teaching-oriented working culture towards open, collaborative RDI processes with different stakeholders that would also remain viable also in exceptional circumstances.

## Footnotes

1 Sales Research Platform (SRP), Next Level Circular and Bioeconomy Actions (CBA), Critical Applied Research of Digitalization in Education (CARDE) and Growth and Learning in Teams (LeGiT).

2 https://www.tuni.fi/en/about-us/tamk/key-information#expander-trigger%2D%2D4f238409-8dc1-44c3-94c0-9ad862fcbf33, accessed 25th of April 2020.
